# Case Report: 18F-PSMA PET/CT Scan in Castration Resistant Prostate Cancer With Aggressive Neuroendocrine Differentiation

**DOI:** 10.3389/fonc.2022.937713

**Published:** 2022-07-22

**Authors:** Marco Bergamini, Alberto Dalla Volta, Irene Caramella, Luisa Bercich, Simona Fisogni, Mattia Bertoli, Francesca Valcamonico, Salvatore Grisanti, Pietro Luigi Poliani, Francesco Bertagna, Alfredo Berruti

**Affiliations:** ^1^ Medical Oncology Unit, Department of Medical and Surgical Specialties, Radiological Sciences, and Public Health, University of Brescia, ASST Spedali Civili di Brescia, Brescia, Italy; ^2^ Department of Molecular and Translational Medicine, Pathology Unit, University of Brescia and ASST Spedali Civili Brescia, Brescia, Italy; ^3^ Nuclear Medicine Unit, Department of Medical and Surgical Specialties, Radiological Sciences, and Public Health, University of Brescia, ASST Spedali Civili di Brescia, Brescia, Italy

**Keywords:** PSMA - prostate specific membrane antigen, neuroendocrine prostate cancer (NEPC), crpc, castration-resistance prostate cancer, small cell prostate cancer, theranostic PSMA radioligands

## Abstract

The development of a neuroendocrine phenotype as a mechanism of resistance to hormonal treatment is observed in up to 20% of advanced prostate cancer patients. High grade neuroendocrine prostate cancer (NEPC) is associated to poor prognosis and the therapeutic armamentarium is restricted to platinum-based chemotherapy. Prostate-specific membrane antigen (PSMA)-based positron emission tomography (PET)/computed tomography (CT) imaging has recently emerged as a potential new standard for the staging of prostate cancer and PSMA-based radioligand therapy (RLT) as a therapeutic option in advanced metastatic castration resistant prostate cancer (mCRPC). PSMA-based theranostic is not currently applied in the staging and treatment of NEPC since PSMA expression on neuroendocrine differentiated cells was shown to be lost. In this case series, we present 3 consecutive mCRPC patients with histologically proven high grade neuroendocrine differentiation who underwent PSMA-PET/CT and surprisingly showed high tracer uptake. This observation stimulates further research on the use of PSMA-based theranostic in the management of NEPC.

## Introduction

Prostate cancer is the most frequent malignancy and the second leading cause of cancer death in Western male population (1). Cancer cells growth and proliferation strongly rely on androgen-androgen receptor (AR) axis. Therefore, androgen deprivation therapy (ADT) is the mainstay of treatment for metastatic prostate cancer (2). The maintenance of ADT in association to taxane-based chemotherapy or next generation hormonal agents (NGHAs) in the castration resistant setting (CRPC) is currently recommended by international guidelines (3). Several mechanisms of resistance to hormonal treatments, such as genomic amplification, activating point mutations and splice variants involving AR have been described (4–8). An increasingly recognized resistance mechanism occurring in up to 20% of advanced prostate cancer involves epithelial plasticity and divergent clonal evolution, in which tumor cells often acquire neuroendocrine features, showing low to absent AR expression (9, 10). Gene expression profiling studies suggest that CRPC following treatment with NGHAs is a heterogeneous disease continuum with distinct phenotypes, based on the expression of AR-regulated and neuroendocrine genes (11, 12). As a matter of fact, a subset of progressive CRPCs shows small-cell carcinoma or neuroendocrine features on metastatic biopsy (13–15).

Prostate-specific membrane antigen (PSMA)-based positron emission tomography (PET)/computed tomography (CT) imaging has recently emerged as a potential new standard for the staging of prostate cancer (16). PSMA is a type II transmembrane glycoprotein highly expressed on prostate cancer epithelial cells (17, 18), especially in high-grade and metastatic castration-resistant disease (19). The expression of FOLH1 gene, encoding PSMA protein, is regulated by AR pathway (20). The induction of lineage plasticity by AR inhibition leads to the suppression of PSMA (21), implying that PSMA-targeted imaging could not effectively visualize neuroendocrine prostate cancers (NEPCs) (22–24). Immunohistochemical and systemic surfaceome profiling studies also indicate that treatment-induced neuroendocrine differentiation is associated with large changes in the repertoire of expressed cell surface proteins (25), with low expression of PSMA and higher expression of neuroendocrine markers, such as synaptophysin, DLL3 and CEACAM5 (14, 26–29). Moreover, PSMA suppression correlates with GLUT12 suppression and glucokinase upregulation, which is positively associated with higher glucose uptake in conventional 18F-fluorodeoxyglucose (FDG) PET imaging (20, 30, 31). These observations suggest a limited utility of PSMA-based theranostic in the management of NEPC (22–24, 32, 33).

On the other hand, a strong 68Ga-PSMA uptake was recently observed in all lesions of a newly diagnosed metastatic small cell prostate carcinoma (34). In addition, PSMA immunostaining positivity was observed in NEPCs after 6 months of neoadjuvant ADT plus enzalutamide (35).

Since May 2021, when PSMA radioligand therapy (RLT) became available in Italy as compassionate use, CRPC patients with disease progression to taxanes and at least one NGHA were offered to perform a PSMA-PET/CT imaging to establish their possible eligibility to treatment with PSMA RLT. In the present paper we report the results of PSMA uptake in 3 consecutive patients who developed a histologically documented high grade NEPC.

## Patients and Methods

**Figure 1:**
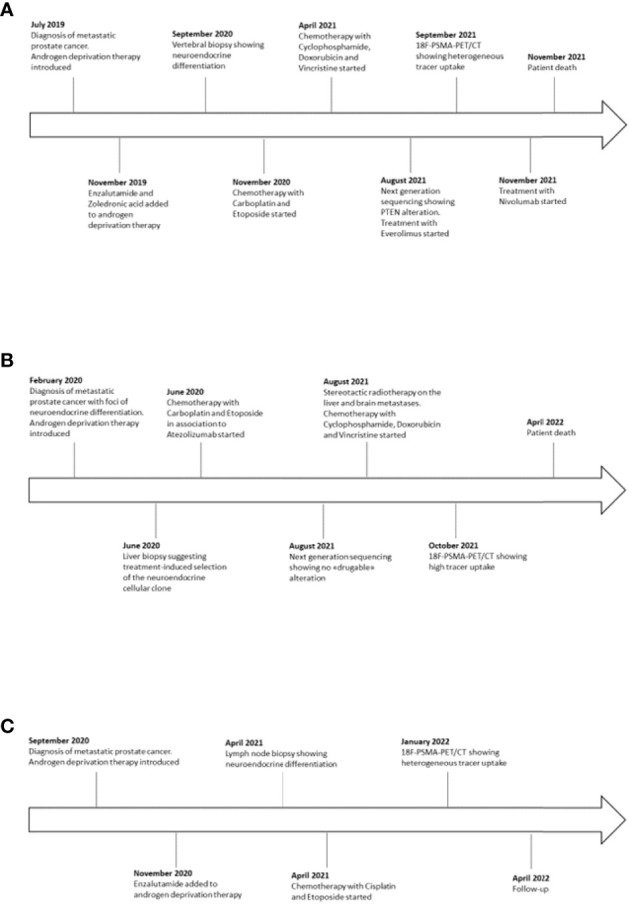
Timeline with relevant data from case 1 **(A)**, case 2 **(B)** and case 3 **(C)**.

From May 2021 to March 2022, 21 CRPC patients with progressing disease to NGHAs and chemotherapy were observed at the Medical Oncology Unit of the ASST Spedali Civili in Brescia (Italy). Three patients (14%) developed an aggressive neuroendocrine phenotype. Graphic timeline of events related to the three cases is displayed in **Figure 1**.

### Case 1

**Figure 2:**
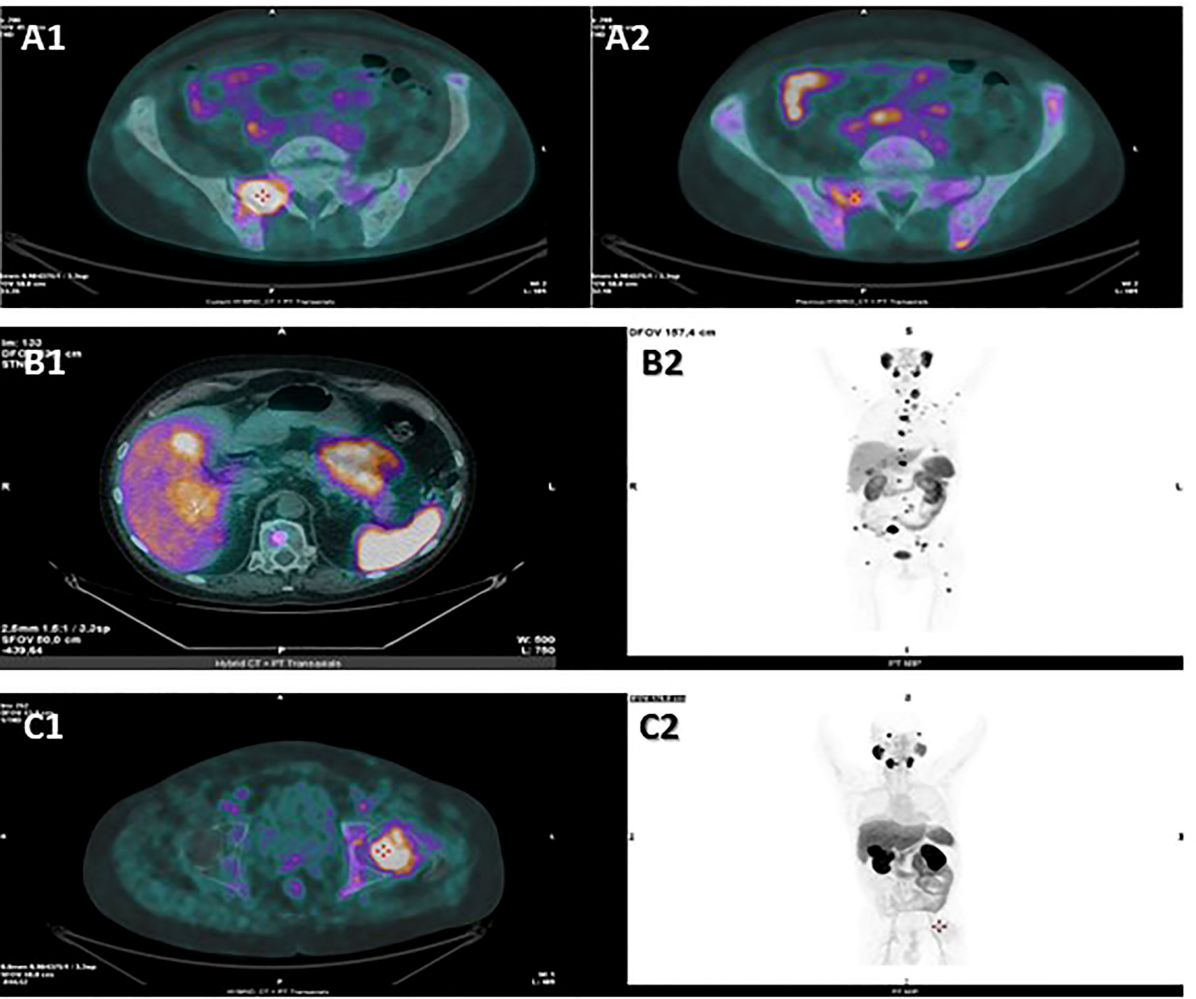
Hybrid CT + PET transaxial images from 18F-FDG PET/CT **(A1)** and from 18F-PSMA-PET/CT **(A2)** examinations performed by patient 1 in September 2021, hybrid CT + PET transaxial **(B1)** and PET MIP total-body **(B2)** images from 18F-PSMA-PET/CT examination performed by patient 2 in October 2021, hybrid CT + PET transaxial **(C1)** and PET MIP total-body **(C2)** images from 18F-PSMA-PET/CT examination performed by patient 3 in January 2022. PSMA-PET scan performed by patient 1 revealed an intense tracer uptake in the prostate and in several bone lesions, such as in the right ala of the sacrum **(A2)**, while the hepatic lesions showed no tracer uptake. The following FDG PET scan revealed high FDG uptake in the liver and in several bone lesions, such as in the right ala of the sacrum **(A1)**. PSMA-PET scan performed by patient 2 revealed high tracer uptake in the liver **(B1, B2)**, bone **(B2)** and lymph nodes **(B2)**. PSMA-PET scan performed by patient 3 showed tracer-avid foci in the prostate and in several osteoblastic lesions, such as in the left femur head **(C1, C2)**, while no uptake was detected in the abdominal lymphadenopathies **(C2)**. CT, computed tomography; PET, positron emission tomography; FDG, fluorodeoxyglucose; PSMA,prostate-specific membrane antigen; MIP, maximum intensity projection.

A 58-year-old man presented with persistent cervical and back pain in July 2019. A spine magnetic resonance imaging (MRI) showed multiple lesions in the cervical, dorsal and lumbar tracts and following L1-L2 vertebral biopsies reported the diagnosis of metastatic prostate cancer. Serum prostate specific antigen (PSA) was 328 ng/ml and prostate biopsies confirmed the diagnosis of Gleason score 5 + 4 prostate adenocarcinoma. ADT with luteinizing hormone-releasing hormone (LHRH)-analogue was then introduced. In November, the patient was enrolled in the BonEnza randomized clinical trial (ClinicalTrials.gov Identifier: NCT03336983) and treatment with Enzalutamide and Zoledronic acid was added to ADT. Serum PSA decreased to 2,1 ng/ml, while CT and whole-body MRI showed a radiological response to therapy. However, in September 2020, due to back pain, a MRI of the spine was performed and showed the appearance of a new vertebral lesion (D5-D6) determining spinal cord compression and dislocation. A biopsy of the lesion was performed and the histological examination revealed metastasis from a combined small cell-large cell neuroendocrine carcinoma. The immunohistochemical staining resulted positive to TTF1 and synaptophysin, Ki67 expression was about 90% and PSA was not expressed. Although serum PSA further decreased to 1,31 ng/ml in response to hormonal treatment, a 18F-FDG PET/CT confirmed disseminated skeletal disease progression. From November 2020 to April 2021, eight Carboplatin plus Etoposide cycles were administered. After an initial partial response to therapy, in April 2021 a 18F-FDG PET/CT revealed several new bone lesions and higher tracer uptake in the pre-existing metastases. After four cycles of chemotherapy with Cyclophosphamide, Doxorubicin and Vincristine, 18F-FDG PET/CT showed skeletal disease progression and the occurrence of two tracer-avid foci in the liver. A next generation sequencing (NGS) according to FoundationOne^®^ platform was performed on the specimen representing NEPC from vertebral lesion, showing RB1 and p53 mutations, TMPRSS2-ERG gene fusion and PTEN splice site alteration, whereas a low tumor mutational burden (TMB) and stable microsatellite status were observed (**Table 1**). Based on the NGS results, a third-line treatment for castration-resistant disease with Everolimus was attempted in August 2021. In order to explore the possibility to undertake PSMA-based RLT, a 18F-PSMA-PET/CT was performed in September 2021, which revealed an intense tracer uptake in the prostate and in several osteoblastic and mixed bone lesions (**Figure 2A2**). However, neither the hepatic metastases nor few of the 18F-FDG-avid bone lesions showed PSMA tracer uptake, suggesting a heterogeneous expression of PSMA across metastatic sites. In detail, the visceral localizations of disease, a typical feature of NEPC, showed high 18F-FDG avidity but no PSMA avidity, hinting at a possible clonal differentiation of disease induced by hormonal treatments. Given the evidence of exclusionary PSMA-negative lesions, the patient was not submitted to PSMA RLT. The following 18F-FDG PET/CT revealed hepatic and skeletal disease progression (**Figure 2A1**) and suggested the onset of secondary localizations of disease in the lungs, a finding subsequently confirmed by a CT examination. After a further line of treatment with Nivolumab inside a clinical trial, the clinical conditions deteriorated and the patient passed away in November 2021.

**Table 1:** Patient 1 and patient 2 genomic signatures and gene alterations detected with next generation sequencing assay FoundationOne CDx.

	SPECIMEN	GENOMIC SIGNATURES	GENE ALTERATIONS
PATIENT 1	Para-vertebral tissue	**Tumor Mutational Burden**: 3 Muts/Mb **Microsatellite status:** MS-Stable	**PTEN:** splice site 165-2A>C **TMPRSS2:** TMPRSS2-ERG fusion **RB1:** loss exons 1-2 **TP53:** H179L
PATIENT 2	Liver	**Tumor Mutational Burden**: 5.04 Muts/Mb **Microsatellite status**: MS-Stable	**NKX2-1**: amplification **RB1**: loss **ERBB2**: amplification - equivocal **KEL**: R516* **TP53**: R283C

Muts, mutations; Mb, Megabase; MS, microsatellite. *Translation termination (stop) codon.

### Case 2

**Figure 3:**
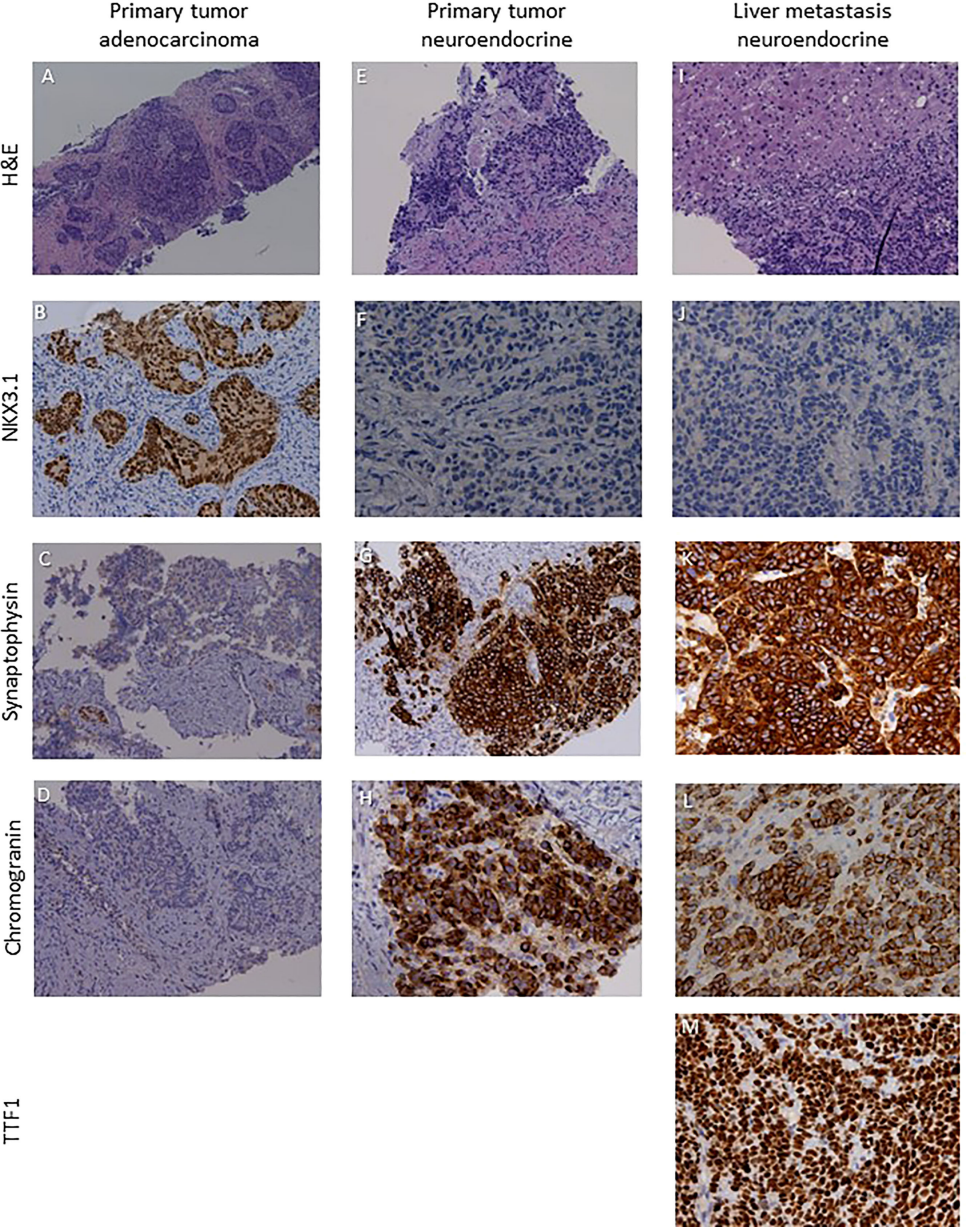
Sections are from patient 2 prostate and liver biopsies and stained as labelled. Primary tumor was a NKX3.1+ synaptophysin- chromogranin-·prostate adenocarcinoma **(A–D)** with foci of NKX3.1- synaptophysin+ chromogranin+ high grade neuroendocrine cardnoma **(E–H)**. Liver metastases were characterized by a NKX3.1- synaptophysin+chromogranin+TTFl+ small cell neuroendocrine phenotype **(I–M)**. Original magnifications: 4X **(A)**, 10X **(B–D, G, I)**, 20X **(E, F, H, J–M)**. H&N, hematoxylin and eosin; TTF1, thyroid transcription factor 1.

A 64-year-old patient was submitted to transurethral resection of bladder (TURB) for a non-muscle invasive bladder neoplasm in 2010. A follow-up cystoscopy performed in February 2020 reported foci of infiltration in the bladder wall. The patient underwent TURB and prostate biopsies, both reporting a Gleason score 4 + 5 prostate adenocarcinoma (**Figures 3A–D**). Additional immunohistochemical staining showed foci of high grade neuroendocrine differentiation (**Figures 3E–H**). Given the nodal metastatic extent of the disease, ADT was introduced. Despite initial biochemical response and volumetric reduction of the lumbo-aortic and pelvic lymphadenopathies, CT and MRI examinations performed in June 2020 showed the appearance of hepatic hypodense lesions, which were histologically characterized as localizations of a TTF1+, synaptophysin+, chromogranin+, PSA-, NKX3.1- small cell neuroendocrine carcinoma (**Figures 3I–M**) with a Ki67 expression of 70%. The histological examination thus hinted at a treatment-induced selection of the neuroendocrine cellular clone previously described in the former prostate biopsy. Five cycles of chemotherapy with Carboplatin and Etoposide in association to Atezolizumab, followed by maintenance therapy with Atezolizumab, were then administered. In the following months, after an initial partial response to therapy, the liver lesions progressed and several new metastatic sites of disease were highlighted in the brain, in the vertebral spine, in the bone pelvis and in the femur. NGS according to FoundationOne^®^ platform was performed on the specimen representing NEPC from hepatic metastasis. The assay revealed RB1 loss, p53 and KEL mutations and NKX2-1 and ERBB2 amplifications. Tumor microsatellite status was stable and the TMB was low (**Table 1**). Since no “druggable” alteration was found, the patient underwent stereotactic radiotherapy on the liver and brain metastases and, in August 2021, a second line of systemic treatment with Cyclophosphamide, Doxorubicin and Vincristine was required. After 6 cycles of chemotherapy, CT and MRI examinations performed in October 2021 showed a partial response on liver metastases. A 18F-PSMA-PET/CT performed in October 2021 revealed high tracer uptake in the bone, lymph node and liver sites of disease (**Figure 2B1, 2**), despite the previously histologically documented neuroendocrine differentiation. Unfortunately, the compassionate use of PSMA RLT had been discontinued pending the drug becoming commercially available and, at last, the patient developed a severe disseminated intravascular coagulation which led to his death.

### Case 3

A 56-year-old man was referred to our Institution for pelvic pain and pollakiuria in September 2020. Serum PSA was 137 ng/ml and prostate biopsies demonstrated a Gleason score 4 + 5 ductal prostate carcinoma. Given the skeletal (pelvis and femur) and nodal metastatic extent of disease shown by bone and CT scan, ADT was introduced and, in November, Enzalutamide was added after the enrollment in the BonEnza clinical trial. A disease response to therapy was obtained and lasted 7 months. In April 2021 a CT examination showed the onset of several lymphadenopathies in the thoracic, abdominal and pelvic regions, although serum PSA levels did not increase (PSA: 0,07 ng/ml). An ultrasound-guided biopsy of an obturator lymph node was performed. The histological and immunohistochemical examinations showed a chromogranin+, synaptophysin+, PSA-, FAP-PSAP-, TTF1- high grade metastatic carcinoma, suggesting the diagnosis of NEPC. Serum neuron-specific enolase (NSE) was 141,9 ng/ml and chromogranin 60 IU/l, supporting the pathologic diagnosis. Eight cycles of chemotherapy with Cisplatin and Etoposide were then administered. Follow-up CTs indicated disease response in lymph nodes and stabilization of bone lesions. Of note, during the course of chemotherapy PSA values maintained undetectable despite the metastatic extent of disease, supporting the diagnosis of a neuroendocrine differentiation of former prostate cancer. In January 2022, a 18F-PSMA-PET/CT was performed, showing tracer-avid foci in the prostate and in several osteoblastic lesions, while no uptake was detected in the abdominal lymphadenopathies (**Figure 2C1, 2**). The heterogeneous expression of PSMA across different metastatic sites was regarded as a clinical contraindication to PSMA-based RLT. At the last visit in February 2022, CT scan showed stable disease and the patient was kept under follow-up.

## Discussion

Treatment induced neuroendocrine differentiation of mCRPC is associated with a deeply divergent transcriptional profile, as compared to classic adenocarcinoma (14), including low-to-absent PSMA expression (24). In our small series, however, a clear positivity to 18F-PSMA radiotracer was observed in three consecutive patients with histologically proven high grade NEPC (with small cell histology in two cases). In two of these patients the heterogeneous PSMA uptake was regarded as a clinical contraindication to PSMA RLT. Instead, one patient showed an intense and homogeneous tracer uptake and was thus considered eligible for PSMA RLT. Unfortunately, the non-immediate availability of the drug and the onset of a serious complication linked to neoplastic progression prevented the RLT administration.

At the best of our knowledge, only rare cases of histologically proven high grade NEPC (34, 36) and one case of presumptive NEPC (37) that resulted PSMA positive at staging are described in the literature.

Derlin et al (38) assessed PSMA theranostic in prostate cancer patients who achieved a neuroendocrine phenotype as assessed by raising serum chromogranin A levels. In this series the outcome of RLT was not adversely influenced by neuroendocrine differentiation, while high PSMA uptake was confirmed to be crucial for achieving a tumor response.

It should be underlined that raising levels of circulating chromogranin A are frequent in patients with CRPC (39). This suggests the presence of a neuroendocrine phenotype which is associated with a worse prognosis (40, 41), but does not imply the development of a high-grade neuroendocrine phenotype, as the cases we have described.

In conclusion, even though the activity of PSMA RLT in high-grade NEPC is still to be documented, our case series suggests that in some of these patients PSMA membrane expression on neuroendocrine differentiated cells is preserved, hinting at a potential role of PSMA theranostic. Given the limited therapeutic options for patients with advanced high-grade NEPC, including potential molecularly driven treatments, we suggest that such patients should not be excluded *a priori* from PSMA-PET/CT testing, as the occasional evidence of high tracer uptake may open the door to PSMA RLT as an additional strategy upon progression to platinum-based chemotherapy.

## Data Availability Statement

All relevant data is contained within the article: The original contributions presented in the study are included in the article/supplementary material, further inquiries can be directed to the corresponding author.

## Ethics Statement

Written informed consent was not obtained from the individual(s) for the publication of any potentially identifiable images or data included in this article.

## Author Contributions

MarB, AD: conceptualization, paper writing IC, FV, SG: paper editing AB: conceptualization, tutoring, paper editing LB, SF, MarB, LP, FB: iconographic support, paper editing. All authors contributed to the article and approved the submitted version.

## Conflict of Interest

The authors declare that the research was conducted in the absence of any commercial or financial relationships that could be construed as a potential conflict of interest.

## Publisher’s Note

All claims expressed in this article are solely those of the authors and do not necessarily represent those of their affiliated organizations, or those of the publisher, the editors and the reviewers. Any product that may be evaluated in this article, or claim that may be made by its manufacturer, is not guaranteed or endorsed by the publisher.
